# Point Cloud Completion Network Based on Multi-Dimensional Adaptive Feature Fusion and Informative Channel Attention Mechanism

**DOI:** 10.3390/s25196173

**Published:** 2025-10-05

**Authors:** Di Tian, Jiahang Shi, Jiabo Li, Mingming Gong

**Affiliations:** Mechanical Engineering College, Xi’an Shiyou University, Xi’an 710065, China; sjh@stumail.xsyu.edu.cn (J.S.); gong_mm@stumail.xsyu.edu.cn (M.G.)

**Keywords:** point cloud completion, feature refinement, attention mechanism, detail reconstruction

## Abstract

With the continuous advancement of 3D perception technology, point cloud data has found increasingly widespread application. However, the presence of holes in point cloud data caused by device limitations and environmental interference severely restricts algorithmic performance, making point cloud completion a research topic of high interest. This study observes that most existing mainstream point cloud completion methods primarily focus on capturing global features, while often underrepresenting local structural details. Moreover, the generation process of complete point clouds lacks effective control over fine-grained features, leading to insufficient detail in the completed outputs and reduced data integrity. To address these issues, we propose a Set Combination Multi-Layer Perceptron (SCMP) module that enables the simultaneous extraction of both local and global features, thereby reducing the loss of local detail information. In addition, we introduce the Squeeze Excitation Pooling Network (SEP-Net) module, an informative channel attention mechanism capable of adaptively identifying and enhancing critical channel features, thus improving the overall feature representation capability. Based on these modules, we further design a novel Feature Fusion Point Fractal Network (FFPF-Net), which fuses multi-dimensional point cloud features to enhance representation capacity and progressively refines the missing regions to generate a more complete point cloud. Extensive experiments conducted on the ShapeNet-Part and MVP datasets compared to L-GAN and PCN showed average prediction error improvements of 1.3 and 1.4, respectively. The average completion errors on the ShapeNet-Part and MVP datasets are 0.783 and 0.824, highlighting the improved fine-detail reconstruction capability of our network. These results indicate that the proposed method effectively enhances point cloud completion performance and can further promote the practical application of point cloud data in various real-world scenarios.

## 1. Introduction

Point cloud completion [1,2,3] aims to recover complete 3D shapes from partial and incomplete point clouds. Complete point cloud data holds significant application value across various domains. In practical scenarios, due to factors such as sensor resolution limitations, occlusions, and reflective surfaces [4,5,6], point cloud is often incomplete, which compromises data integrity and affects the performance of subsequent processing. Therefore, recovering a complete output from partial input has become a pressing challenge [7,8]. For instance, in autonomous driving, accurate 3D environmental perception is critical to ensuring the safe operation of vehicles. Point cloud completion techniques can enhance the accuracy and robustness of 3D perception, enabling autonomous systems to better recognize and interpret their surroundings, thus facilitating more intelligent and safer decision-making. Moreover, point cloud completion [9,10,11] serves as a crucial step toward achieving high-precision 3D model reconstruction. It contributes to generating more complete and accurate 3D models, providing strong support for subsequent analysis and application.

The current mainstream point cloud completion methods are mostly based on deep learning frameworks, using encoder-decoder structures to extract potential features from incomplete inputs, and then generate complete point clouds through decoding. The focus of the point cloud completion task is to repair surface details of objects, mainly involving research on small surface details and surface continuity reconstruction. The method proposed in this article focuses more on the reconstruction of surface continuity, while also focusing on the restoration of local features and surface details of objects. Therefore, this study defines fine-grained as surface continuity [12,13]. The existing mainstream point cloud completion methods focus on the global features of the object when extracting features, and have a slight deficiency in capturing local features of the object. Meanwhile, in the process of generating a complete point cloud, the control over object details is not sufficient, resulting in the need to improve the detailed features of the point cloud after completion, which reduces the integrity of the point cloud data. The fundamental reason for this problem is, on the one hand, the unstructured and irregular nature of point cloud data, which poses challenges for the direct application of convolutional neural networks in point cloud processing. Although methods like PointNet [14] and PointNet++ [15] have partially addressed these challenges, their modeling of local topological relationships between points remains insufficiently refined, often overlooking high-frequency geometric information within local structures. On the other hand, many completion models are architecturally biased toward global semantic modeling [16,17,18]. Even when local feature modules are introduced, they often fail to effectively integrate multi-scale contextual information or complex geometric constraints, making it difficult to accurately capture detail-rich or geometrically complex local regions.

Furthermore, the lack of effective constraints on fine-grained features not only compromises the visual quality of point cloud completion [19,20,21,22], but also negatively impacts downstream tasks that rely heavily on local detail. For instance, in autonomous driving, the accuracy of point cloud completion directly affects the recognition of object boundaries; erroneous completions may lead to misdetections in object recognition tasks [23,24]. Similarly, in robotic grasping or human–machine interaction scenarios, the precision of surface feature recovery is closely tied to the feasibility of motion planning and the safety of physical operations.

With the rapid development of deep learning techniques [25,26], an increasing number of studies have leveraged neural networks to tackle the point cloud completion problem, improving the quality of shape reconstruction. Representative works such as LGAN-AE, PCN, and 3D-Capsule adopt end-to-end encoder–decoder frameworks [27], taking incomplete point clouds as input and generating semantically consistent and structurally complete 3D point clouds as output. These approaches have achieved remarkable results in recovering overall shapes, particularly excelling in capturing global contours and incorporating category-level priors, which has greatly enhanced the automation and scalability of the completion process. However, these methods also share certain limitations. On the one hand, they tend to prioritize global semantic consistency during modeling, while overlooking geometric details and structural integrity in local regions, leading to missing or blurred fine-grained features in the generated results. On the other hand, many of these models lack mechanisms for fine-grained selection and enhancement of key features, which limits their ability to represent complex geometric structures. For example, LGAN-AE primarily improves the realism of global structures through adversarial learning, but lacks sufficient modeling of local details. PCN introduces a multi-stage generation strategy, yet still falls short in recovering high-frequency geometric features. 3D-Capsule incorporates capsule networks to enhance spatial feature awareness, but remains insufficient in modeling local deformations and edge features. As a result, while the generated shapes may closely resemble real objects at a macro level, discrepancies persist in the microstructure, making it difficult to meet the demands of fine-grained modeling and high-precision perception in real-world applications.

To address the aforementioned limitations, this paper first designs a multi-level, multi-resolution farthest point sampling strategy, SCMP, which performs key point sampling followed by local grouping and feature extraction operations. This approach effectively preserves structural details and constructs point cloud representations with rich local semantics, providing high-quality inputs for subsequent completion processes. In addition, we propose SEP-Net, a strategy that combines channel attention mechanisms with pooling operations. By suppressing redundant features and enhancing key responses, SEP-Net refines feature representations and significantly improves the network’s capability in reconstructing detailed structures, thereby enhancing the overall point cloud completion performance. Building on these components, we propose a novel point cloud completion network called FFPF-Net, which integrates the SCMP module and SEP-Net to improve local feature extraction and enhance detail modeling. Specifically, the SCMP module is employed during the point cloud processing stage to extract a representative subset from the raw point cloud. This allows the network to represent point cloud data in a more compact and abstract manner by selecting key points, thus reducing data volume, lowering computational complexity, and improving processing efficiency while retaining informative structural cues. The SEP-Net module, a powerful architecture that combines attention mechanisms with pooling layers, is applied during the feature processing stage. It aims to explicitly model the interdependencies between feature channels to enhance the network’s sensitivity to critical features. Starting from the limitations of existing point cloud completion methods in modeling local features and restoring fine details, this paper focuses on improving feature representation accuracy and structural completeness. We propose a point cloud completion approach based on multi-dimensional adaptive feature fusion and informative channel attention mechanisms, which significantly enhances the detail quality of the completed point clouds.

The main contributions of this work are summarized as follows:

(1) To address the problem of losing local structural information, we propose the SCMP module for feature extraction and fusion of the input point cloud. It performs multi-level feature extraction on the raw point cloud, effectively capturing local features and reducing the loss of fine-grained detail information.

(2) To overcome the insufficient ability to reconstruct fine details, we introduce the SE-Net module, which enhances the interdependence among features and adaptively adjusts their weights. This significantly improves the network’s capability in modeling locally complex structures during the completion process.

(3) Based on the proposed SCMP and SEP-Net modules, we further develop a Feature Fusion Point Fractal Network (FFPF-Net) for point cloud completion. Extensive experiments on the ShapeNet-Part and MVP datasets demonstrate the feasibility and effectiveness of FFPF-Net in completing point cloud data.

## 2. Related Work

### 2.1. End-to-End Deep Learning-Based Point Cloud Completion Methods

With the development of 3D perception technology, deep learning-based point cloud completion methods have become the mainstream direction. Early research primarily relied on voxel representations or mesh reconstruction, converting point clouds into regular grid formats to facilitate neural network processing. However, these approaches suffer from significant limitations in computational efficiency and spatial resolution. In recent years, with the advancement of the PointNet series, end-to-end completion models that directly process raw point clouds have emerged, greatly improving the performance of 3D completion tasks.

LGAN-AE [28] is an autoencoder structure based on generative adversarial networks, capable of effectively learning the latent distribution of point clouds and enhancing the structural consistency of completion results through adversarial loss. However, this method focuses more on global shape generation and insufficiently models local geometric details, resulting in weaker performance at the detail level. PCN [29] extracts global features via an encoder and generates complete point clouds through a staged decoder. Its coarse-to-fine two-stage strategy achieves notable results in reconstructing overall contours, but due to a lack of fine-grained modeling of local structures, the recovered details remain insufficient. The 3D-Capsule [30] network introduces a capsule network mechanism aiming to model spatial relationships and dynamic routing of features in point cloud data, thereby enhancing the network’s geometric understanding capability. Nonetheless, its ability to model complex detailed structures is still constrained by the granularity of local modeling inherent to the network architecture. Although the aforementioned deep learning-based point cloud completion [31,32] methods have made progress in overall shape reconstruction, their heavy reliance on global features often leads to inadequate capture of local geometric details. This is especially problematic in scenes with complex edges, non-uniform densities, or detail-rich structures, where the completion results tend to be blurred or structurally degraded. These limitations have prompted researchers to focus on the importance of local structural information in the completion process and to explore how to explicitly incorporate local feature modeling mechanisms to enhance the network’s and restore spatial details.

### 2.2. Point Cloud Completion Methods Based on Local Feature Modeling and Detail Reconstruction

Enhancing a model’s perception and modeling capability of local structures has long been a key challenge in cloud tasks. Traditional processing methods, PointNet++, extract local features through hierarchical sampling and neighborhood aggregation. However, their use of fixed-radius or k-nearest neighbor (k-NN) strategies for defining local regions struggles to adapt to the inherent irregularity and non-uniform distribution of point clouds, resulting in the omission of some edge information and high-frequency geometric details.

To improve the perception of local geometric structures, some studies have attempted to incorporate graph neural networks (GNNs) or attention mechanisms. For example, DGCNN [33] dynamically constructs KNN graphs and extracts edge features within these graphs to enhance the modeling of local geometric relationships. PointTransformer [34] employs spatial-channel self-attention mechanisms to learn dependencies between points, thereby boosting the expressiveness. These methods alleviate the loss of local structural information to some extent but involve complex designs with high computational costs, and they have not been specifically optimized for modeling geometric details in missing regions within completion tasks. To address the issue, some works have proposed completion mechanisms that combine multi-scale feature fusion with structural guidance. For instance, SnowFlakeNet [35] adopts a layer-wise “snowflake” point generation approach that gradually refines local details from coarse structures. However, its detail enhancement still depends on the prior distribution from coarse layers, making it difficult to directly capture authentic details from the raw input. Although local feature modeling techniques have improved detail recovery in point cloud completion to some degree, relying solely on fixed-range local operations remains limited when facing large-scale structural variations or scenarios where local and global semantics are strongly correlated. This results in constrained receptive fields and insufficient contextual understanding. To further enhance the modeling capability of key information, attention mechanism-based methods have gradually become a research focus in recent years.

### 2.3. Attention Mechanism-Based Point Cloud Completion Methods

The introduction of attention mechanisms has significantly alleviated the limitations of traditional point cloud completion methods in modeling local details. By dynamically adjusting the response weights of different spatial positions or feature channels, attention mechanisms enable models to focus more effectively on key regions and important shape features within point clouds, thereby improving geometric accuracy and structural fidelity in completion results.

For example, PoinTr [36] is the first work to apply the Transformer architecture to point cloud completion. It models long-range dependencies between points using learnable query vectors and self-attention mechanisms, effectively enhancing the model’s understanding of global context. However, its ability to recover fine-grained geometric details remains limited. To further improve local structure perception, AnchorFormer [37] proposes a Transformer framework based on spatial anchor partitioning. By aggregating local regions and performing attention modeling within subregions, it reduces computational complexity while strengthening the modeling of critical areas. SeedFormer [38] employs a patch seed structure combined with an upsample transformer, first generating a sparse skeleton and then performing point-level upsampling via attention mechanisms. This guides the model to focus on structural core regions, improving detail restoration. SVDFormer [39] goes a step further by integrating geometric principal axis information extracted through singular value decomposition (SVD) with self-attention mechanisms. This guides the network to adaptively adjust feature responses based on global structures, enhancing the identification and reconstruction of local details while maintaining overall consistency. In summary, attention mechanisms have become an effective modeling tool in recent point cloud completion research, significantly enhancing the ability to capture critical information and mitigating deficiencies in local detail modeling to some extent. Nevertheless, our study finds that current methods still face limitations in fine-grained feature modeling, computational efficiency control, and adaptability to density variations. To address these issues, this paper refines missing point clouds through multi-dimensional feature fusion, aiming to generate more complete point cloud models.

### 2.4. Dataset Introduction

Current point cloud completion research primarily relies on core datasets such as Shapenet-Part, MVP, and FootGait3D. The Shapenet-Part dataset is a large-scale, multi-category 3D model database covering dozens of common object categories, such as chairs and cars.

Each category contains a vast number of 3D models, which include geometric information, topological structures, and rich descriptive data such as labels and categories, making it highly suitable for 3D shape understanding and analysis. Therefore, it is ideal for local reconstruction tasks that require detailed modeling of specific structures. The MVP dataset is a multi-view partial point cloud dataset containing over 100,000 high-quality scans. This dataset presents partial 3D shapes from 26 uniformly distributed camera poses for each 3D CAD model, primarily used for point cloud completion and registration research. The MVP dataset integrates a wide range of categories and a large number of 3D model point cloud data, covering various objects and scenes. Additionally, each 3D CAD model is scanned from 26 uniformly distributed camera poses, ensuring data diversity and high quality. Therefore, it is suitable for detailed 3D reconstruction tasks. FootGait3D, as an innovative dataset in the medical field, specifically collects motion point cloud data of the ankle and foot region. It addresses common occlusion issues in medical imaging through precise annotation, opening up new avenues for rehabilitation medicine and motion analysis. Furthermore, the emerging PointTr Benchmark provides an important testing platform for the development of geometric perception Transformer models by collecting point cloud data from complex real-world scenes such as streets and indoors. And KITTI, as a classic dataset in the field of autonomous driving, continues to provide a verification benchmark for sparse point cloud completion tasks around vehicles. These datasets, spanning from basic research to vertical fields such as healthcare and autonomous driving, collectively form a comprehensive verification system for point cloud completion technology, driving rapid development in this field. Considering that the research content of this paper focuses on the restoration of detailed features and precise completion of missing areas, Shapenet-Part and MVP are selected for experiments in subsequent sections.

## 3. Method

Based on the aforementioned issues, this paper proposes the SCMP feature extraction and fusion module and the SEP-Net feature refinement module. Building upon these, we further introduce the FFPF-Net point cloud completion network, which addresses the shortcomings in local feature extraction and detail reconstruction. The proposed approach enhances the local feature representation and detail recovery capabilities of point cloud completion, while improving overall feature representation. This section presents a detailed introduction to the methods proposed in this paper

### 3.1. Overall Framework

During the point cloud data acquisition process, point loss is inevitably caused by factors such as the characteristics of the tested objects, processing methods, and environmental influences. The main reasons for this loss include specular reflection, signal absorption, and occlusion by external objects. After data acquisition, steps such as denoising, smoothing, and registration are also required, which can further lead to point loss.

To more effectively handle data and accomplish the completion task, this paper applies the SCMP module and SEP-Net module during the point cloud processing and feature processing stages, respectively. The SCMP module extracts a representative subset from the raw data, representing the data in a more compact and abstract manner. By selecting key points, it reduces the number of points, thereby lowering computational complexity and improving processing efficiency, while obtaining point cloud data containing richer information. The SEP-Net is an attention mechanism-based convolutional neural network architecture applied in the feature processing stage. It explicitly models the interdependencies between feature channels to enhance the network’s sensitivity to important features.

**Figure 1:**
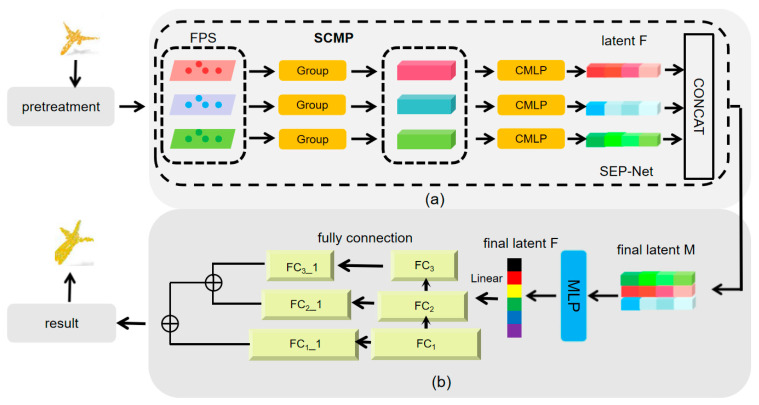
The FFPF Net structure diagram of the point cloud completion network proposed in this article. (**a**) Multilayer perceptron feature extraction structure. (**b**) Fully connected decoding structure.

Based on the SCMP module and SEP Net module, we constructed the Feature Fusion Point Cloud Completion Network (FFPF Net), whose overall architecture is clearly shown in Figure 1.

Firstly, in section A of the structure, the input raw point cloud data is sent to the SCMP module for preliminary processing. This module can generate three point cloud datasets with different characteristics according to their different composition. Subsequently, with the collaborative effect of the SA module, these point cloud datasets were further enriched, containing more point cloud information. Next, we use three independent CMLP components to map these three different scales of point cloud data into three independent composite latent vectors. These vectors represent the key features extracted at specific resolution levels from point cloud data.

Next, these informative vectors are fed into the SEP Net module for deep processing. Within this module, information exchange between feature channels is achieved through the application of SE Net attention mechanism and the execution of pooling operations. During this process, adaptive attention weight calculations were performed on each channel of the input feature map, thereby enhancing the expression of important features and effectively aggregating the feature map locally, ensuring the complete preservation of the main features.

Subsequently, in section B of the structure, the feature vectors processed by the SEP Net module are sent to the decoder for further decoding operations. The decoder consists of a series of fully connected layers that take feature vectors as input and gradually parse out three key features: F1, F2, and F3. Among them, F3 obtained the prediction result Yp (dimension M1 × 3) of the main center point through the processing of convolutional layers. On this basis, combined with the feature information of F2, we further calculated the relative coordinates Ysec of the secondary center. Finally, utilizing the combined effect of F1 and Ysec, we generated the final point cloud data Yd. It is worth noting that Yd also attempts to accurately match feature points sampled from real values to ensure the accuracy and authenticity of the completion results.

Due to the multi-scale generation architecture adopted by FFPF Net, high-level features can have a profound impact on the expression of low-level features. At the same time, low resolution feature points can effectively propagate local geometric information to high-resolution prediction results, thereby achieving comprehensive and accurate completion of point cloud data.

### 3.2. Subsection Scmp Module

Current point cloud completion methods often suffer from insufficient local feature capture and low precision in detail reconstruction. Existing approaches struggle to accurately capture local geometric features and texture details within point clouds, primarily due to the inherent sparsity, irregularity, and noise present in data. This complexity makes local feature extraction challenging, and inadequate capture of local features leads to significant differences in details between the completed and original point clouds, adversely affecting the overall visual quality and subsequent applications. Similarly, detail reconstruction is a critical step in the point cloud completion process. However, existing methods often find it difficult to precisely reconstruct fine details while maintaining global structure. Low accuracy in detail reconstruction results in completed point clouds that appear visually unrealistic or coarse, thereby impacting user experience and downstream processing.

**Figure 2:**
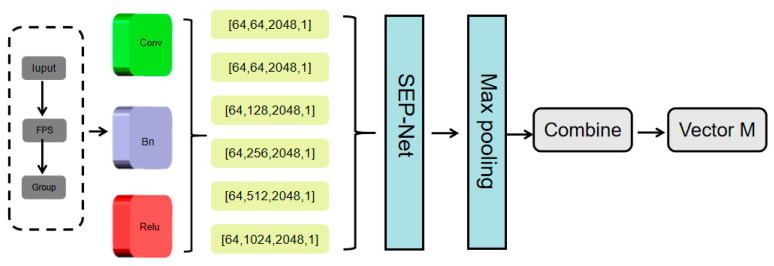
The SCMP module for feature processing mentioned in this article.

To address issues, this paper proposes a feature extraction and fusion module (SCMP), which combines Set Abstraction and Composite Multi-Layer Perceptron (CMLP) to enhance point cloud feature acquisition and reduce the loss of local details. The architecture of the SCMP module is illustrated in Figure 2.

The SCMP module first selects a series of key points from the original point cloud using Farthest Point Sampling (FPS), which iteratively chooses the point farthest from the already selected set as a new key point until the predetermined number of key points is reached. Input point cloud $P=\left\{p_{1}{,p}_{2}{,p}_{3}\cdots \cdots \right.p_{N}\}$, the number of sampling points is *N*, the sampled point set is ${S=\{s}_{1}{,s}_{2}{,s}_{3},\cdots \cdots \left.s_{M}\right\}$, the sampled point set is *M*. Then, points from the original point cloud are assigned to local regions centered on each key point using K-Nearest Neighbors (KNN). For each key point, KNN finds the nearest K points and assigns these points to the group corresponding to that key point. The specific formulation is as follows:

(1) $$G_{i}=\{p_{j}\in P\left|d\left(s_{i},p_{j}\right)\right.\leq r\}$$

In Equation (1), *G_i_* is a group, d *(***s***_i_*, **p***_j_)* is the distance between the two, *r* is the search radius.

Then, feature extraction is performed by extracting features from each local region to represent the information of that region. For each local region, MLP and pooling operations are applied to the points to extract features. The MLP typically consists of multiple fully connected layers that can learn complex feature representations. The pooling operation produces a fixed-length feature vector representing the region’s information. Feature extraction can be expressed by Equation (2).

(2) h1=ReLU(W1Gi+b1)h2=ReLU(W2Gi+b2)   ⋯⋯fi=WLhL−1+bL

In Equation (2), **W***_l_*, **b***_l_* are the weights and biases of the *l*-th layer, *L* is the number of layers in MLP, *ReLU* is activation function, h_*i*_ is hierarchical feature, **f***_i_* is a feature vector.

**Figure 3:**
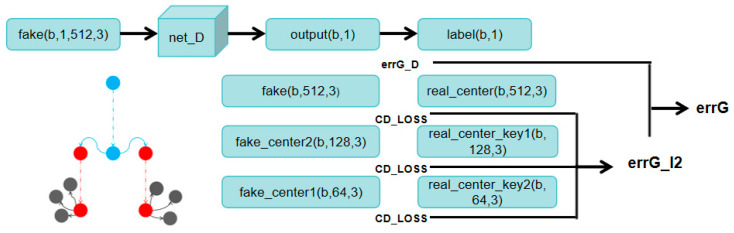
The FCD module for point cloud generation mentioned in this article.

SCMP can extract features from point clouds layer by layer, with the center points obtained from each layer being a subset of the center points from the previous layer. The input point cloud is processed through the SCMP module, and a more informative point set is obtained by uniformly sampling the input point set. The resulting downsampled point cloud and the original point cloud are separately input into the Combined Multi-Layer Perceptron. In the Composite Multi-Layer Perceptron, we use MLP to encode points into multiple dimensions, then apply max pooling to the outputs of the last four layers of MLP to obtain multidimensional feature vectors. These vectors are then combined to form the final feature vector. The final feature vector is used as input to the FCD module, which generates a complete point cloud to represent the missing areas in the region, as shown in Figure 3. The foundation of the FCD is a fully connected decoder, which can predict the global geometric shape of point clouds very effectively. However, it only uses the last layer, it may cause a loss of local geometric information. To address this, we adopt a multi-level structure. First, the feature vectors are passed through a fully connected layer, each responsible for predicting point clouds at different resolutions. The main center point is predicted starting from the deepest feature layer, followed by the prediction result of the center point in the second layer. The relevant coordinates of each point are generated by combining the previous points as centers and the extracted features. Finally, the complete point cloud is obtained.

Based on the above analysis, SCMP performs fine layered feature extraction on point cloud data, which can flexibly integrate multi-scale features, effectively address the challenges of point cloud data with varying densities. This module accurately captures the subtle features of point clouds by performing feature extraction operations at different scales and cleverly fusing these multi-scale features. Furthermore, SCMP not only focuses on multi-scale features but also excels in deeply mining multi-layer features. This multi-level and multi-dimensional feature extraction mechanism enables SCMP to comprehensively understand the complex structure of point clouds, accurately capture the rich geometric and semantic information contained therein. Therefore, SCMP not only improves the problem of insufficient local feature extraction but also lays a solid, comprehensive foundation for subsequent tasks, enhancing the accuracy and precision of the completion effect.

### 3.3. Sep-Net Module

**Figure 4:**
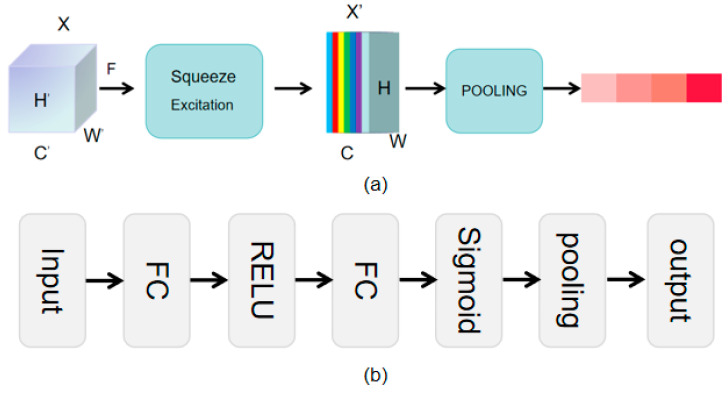
The feature refinement SEP Net module proposed in this article. (**a**) SEP-Net architectural feature map. (**b**) SEP-Net operational flowchart.

The SEP-Net module explicitly models the dependency relationships between convolutional feature channels by introducing the “Squeeze-and-Excitation” (SE) block, thereby significantly improving network performance with almost no increase in computational cost. Secondly, it automatically obtains the importance level of each feature channel through learning. This “feature-calibration” strategy enhances the network’s representational ability. SEP-Net, as shown in Figure 4a, mainly includes three key operations: Squeeze, Excitation, and Scale.

The purpose of the squeezing operation is to encode the global spatial features of each channel into a global feature, usually achieved through Global Average Pooling (GAP). For an input feature map **X**, its shape is (*H*, *W*, *C*), where *H* and *W* are the height and width of the feature map, respectively, and *C* is the number of channels. The squeezing operation performs global average pooling on each channel, resulting in a vector of length *C*, denoted as **z**. The specific calculation formula is as follows:

(3) $$z_{c}=\frac{1}{H\times W}\sum_{i=1}^{H}\sum_{j=1}^{W}X_{c}\left(i,j\right)$$

In Equation (3), **X***_c_*(*i*, *j*) indicate the value of the *c*-th channel in feature map **X** at position (*i, j*), *H* and *W* are the width and height of the feature map, and **z***_c_* is the feature vector of length *c*.

The purpose of the excitation operation is to learn the nonlinear relationships between various channels through a simple fully connected neural network, in order to obtain the weights of each channel. This fully connected neural network typically consists of two fully connected layers. The first fully connected layer is used for dimensionality reduction, reducing the number of parameters and computational complexity. The second fully connected layer is used for dimensionality enhancement, restoring the original number of channels. Finally, the weight of each channel is obtained through the sigmoid activation function, denoted as **s**. The specific calculation formula is as follows:

(4) $$s=\sigma (W_{2}\delta (W_{1}z))$$

In Equation (4), **W**_1_ and **W**_2_ are the weights of two fully connected layers, **z** is the feature vector, *δ* is the ReLU activation function, *σ* is a sigmoid activation function, **s** is the weight of each channel.

The purpose of the recalibration operation is to apply the channel weights obtained from the excitation operation to the original feature map, in order to achieve recalibration of the feature map. The specific operation is to multiply the weight **s** with each channel of the original feature map **X** through channel multiplication to obtain the final feature map **X**′. The calculation formula is as follows:

(5) $$X_{c}^{\prime }=s_{c}\cdot X_{c}$$

In Equation (5), **X**^′^*_c_* and **X***_c_* represent the *c*-th channel of the recalibrated and original feature maps, respectively, and **s***_c_* is the weight of this channel.

Given the input features, after a series of convolution operations, new features are obtained, as shown in Figure 4b. After performing a global average pooling operation on the new features, and passing them through two fully connected layers and two activation functions, the final output feature map is obtained. After the first FC layer, the ReLU activation function is applied to output a feature map of size 1× 1× C. After the second FC layer, the Sigmoid activation function is used to normalize the values of each layer to the range (0,1), representing the weight of each channel. The output feature size of the second FC layer is 1 × 1 × C. Finally, the weights are multiplied to obtain the output feature map.

The SEP-Net module uses a highly adaptive mechanism to accurately grasp the importance of each channel’s contribution to the overall task. This module is dedicated to fine weight distribution of feature channels to achieve effective enhancement of key information, effective suppression of redundant information. The SEP-Net module applies these learned weight values to the corresponding channels of the original feature map to achieve weighted adjustment of features. In this way, useful channel features are significantly enhanced, while unimportant channel features are effectively suppressed. This adaptive weight allocation mechanism not only improves the sensitivity of the model to key information but also reduces the interference of redundant information on model performance, thereby enhancing the ability to reconstruct details of missing point clouds.

## 4. Experiment

To verify the effectiveness in this article, experiments were conducted on both the Shapenet-Part dataset and the MVP dataset in this section. The Shapenet-Part is a 3D model database, such as chairs, cars, etc. Each category contains a large number of 3D models, which include geometric information, topological structures, and rich descriptive data such as labels and categories, making them very suitable for 3D shape understanding and analysis. The MVP dataset is known for its large-scale and multi-perspective characteristics. It contains a large number of residual cloud samples, providing abundant resources for point cloud learning tasks. Meanwhile, the multi-perspective feature enables the model to learn the shape and structure of objects more comprehensively, improving the quality of point cloud completion.

### 4.1. Shapenet-Part Dataset Experiment

#### 4.1.1. Shapenet-Part Comparison of Completion Effect Experiments

In this section, we compare our method, including L-GAN [28], PCN [29], 3D PointCapsule [30], CGCN [31], and DAF-SAC [32]. Networks. Since the existing methods are trained on different datasets, we retrain them on the same dataset for quantitative evaluation. All methods do not receive any additional information. We use two evaluation metrics: Pred → GT (Error from predicting point cloud to actual point cloud) and GT → Pred (Error from real point cloud to predicted point cloud). The results are shown in Table 1. To ensure that our evaluation is reasonable, we also calculated the Pred → GT error and GT → Pred error on the missing areas. The results are shown in Table 2.

As shown in Table 1 and Table 2, among the 13 categories tested, in terms of the average point cloud completion results Pred → GT/GT → Pred for the overall point cloud, LGAN-AE is 1.869/1.615, PCN is 1.802/1.662, 3D Capsule is 1.927/1.713, CGCN is 1.268/1.156, and DAF-SAC is 1.001/0.975. In the completion results of the missing point cloud, the LGAN-AE average value is 5.395/2.603, the PCN average value is 4.360/2.661, the 3D Capsule average value is 5.829/3.008, the CGCN average value is 3.079/2.905, and the DAF-SAC average value is 2.480/2.239. However, the average values of the method proposed in this paper are 0.579/0.475 and 2.482/2.140, respectively. Experimental results show that our method has good advantages. The results in Table 1 and Table 2 indicate that our method can generate more accurate point clouds while having less distortion in the overall point cloud and the missing area point cloud.

#### 4.1.2. Shapenet-Part Dataset Robustness Test

This article conducted robustness tests on the Airplane, Guitar, and Skateboard classes in the Shapenet-Part dataset. In the robustness testing, we not only changed the M parameter in FCD to control the number of output points of the network, but also trained the network to repair shapes with different degrees of incompleteness, in order to evaluate its stability and recovery ability in the face of missing or damaged input data. By adjusting the M parameter, we can simulate point cloud data with varying degrees of sparsity, then test the performance of the network in repairing these incomplete shapes. The experimental results are shown in Table 3.

As shown in Table 3, 25%, 50%, and 75% represent the degrees of point cloud loss. When the Missing Ratio is 25%, the missing rates for the three are 0.784/0.520, 0.683/0.493, and 0.803/0.658, respectively. When the Missing Ratio is 50%, the missing rates for the three are 0.763/0.531, 0.668/0.547, and 0.824/0.603, respectively. When the Missing Ratio is 75%, the missing rates for the three are 0.792/0.594, 0.694/0.552, and 0.795/0.656, respectively. The average input error for 25% is 0.757, for 50% it is 0.752, and for 75% it is 0.760. The input errors for all three parts are around 0.76. This indicates that the network in this article has strong robustness in handling point clouds with different degrees of missing data, can stably recover the missing point clouds.

#### 4.1.3. Shapenet-Part Comparison Experiment of Dataset Cd

**Figure 5:**
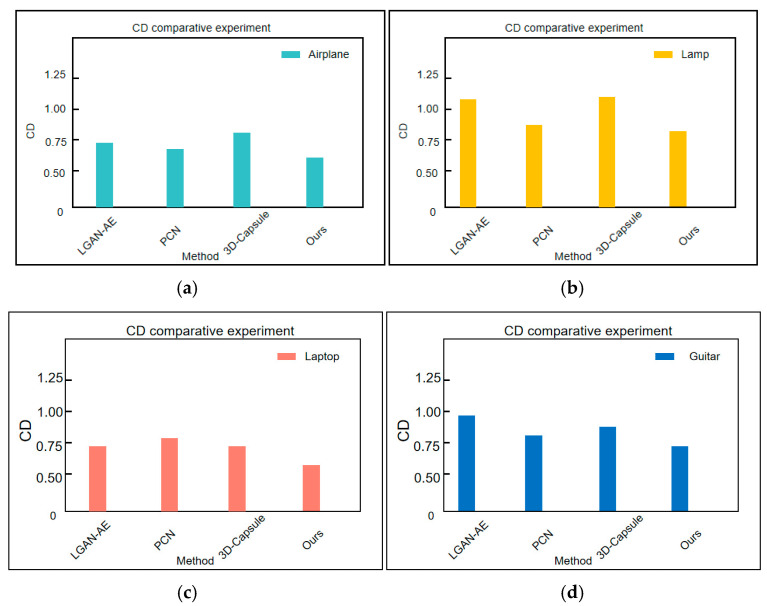
Shapenet-Part dataset CD comparative experiment. (**a**) Airplane CD comparative experiment. (**b**) Lamp CD comparative experiment. (**c**) Laptop CD comparative experiment. (**d**) Guitar CD comparative experiment.

This paper conducts CD comparative experiments on the Airplane and Lamp categories from the Shapenet-Part dataset, with the results illustrated in Figure 5. A larger CD value indicates greater dissimilarity between the two point clouds, while a smaller distance signifies better reconstruction performance.

To further validate the effectiveness of the proposed method, this paper conducted CD comparison experiments on the Airplane, Lamp, Laptop, and Guitar categories of the Shapenet Part dataset. The experimental results are shown in Figure 5. Compared with LGAN-AE, the CD values of the proposed method in this paper decreased by 0.21, 0.15, 0.17, and 0.22 in the categories of Airplane, Lamp, Laptop, and Guitar, respectively; Compared to PCN, the CD values decreased by 0.13, 0.13, 0.15, and 0.12, respectively; Compared to 3D capsules, the CD values decreased by 0.22, 0.16, 0.18, and 0.14, respectively. These data show that the method proposed in this article significantly outperforms other mainstream methods in terms of CD values, with average reductions of 20%, 15%, 13%, and 17%, respectively.

#### 4.1.4. Shapenet-Part Dataset Ablation Experiment

To demonstrate the effectiveness of the proposed SCMP and SEP-Net modules and to gain a deeper understanding of the internal mechanisms of the model for optimizing its structure, a series of carefully designed ablation experiments were conducted on the ShapeNet-Part dataset. These experiments systematically removed or replaced the SCMP and SEP-Net modules to observe and analyze changes in model performance, thereby accurately evaluating the contribution of each module to the overall performance. The results of the ablation studies not only validate the effectiveness of but also provide valuable guidance for further optimizing the model architecture, ensuring its outstanding performance on the ShapeNet-Part dataset. The results are shown in Table 4.

As shown in Table 4, the proposed modules all lead to a reduction in the Chamfer Distance (CD) value. When using only the SCMP module, the CD value decreases by 0.011; when using only the SEP-Net module, the CD value decreases by 0.037. When both modules are used together, the CD value decreases by 0.055. Therefore, the SCMP and SEP-Net modules proposed in this paper have been experimentally verified to reduce the CD value by approximately 5%, enhancing the quality of point cloud features, effectively reducing the loss of geometric information, and strengthening the inter-feature dependencies, thereby improving the feature representation capability.

### 4.2. Mvp Dataset Experiment

#### 4.2.1. Mvp Comparison of Dataset Completion Effects

To avoid the incompleteness of experimental results obtained from a single dataset, we also conducted the same experiment on the MVP dataset. Compared to the Shapenet-Part dataset, the MVP dataset first collects point cloud data from a wide variety of 3D models, covering various objects and scenes. Secondly, each 3D CAD model is scanned from 26 evenly distributed camera poses, ensuring data diversity and high quality. The results are shown in Table 5 and Table 6. In the 13 categories of the MVP dataset, the testing error of the method proposed in this paper is lower than that of other mainstream methods, and it can obtain higher precision point clouds with smaller distortion during the generation of missing point clouds.

As shown in Table 5 and Table 6, among the 13 categories tested, in terms of the average point cloud completion results Pred → GT/GT → Pred for the overall point cloud, LGAN-AE is 2.721/2.583, PCN is 2.778/2.926, 3D Capsule is 2.781/2.893, CGCN is 1.846/1.801, and DAF-SAC is 1.671/1.601. In the completion results of missing point clouds, the LGAN-AE average value is 6.626/4.554, the PCN average value is 5.966/4.390, the 3D Capsule average value is 6.895/4.811, the CGCN average value is 4.080/3.869, and the DAF-SAC average value is 3.481/3.301. However, the average values of the method proposed in this paper are 1.543/1.651 and 3.473/3.04, respectively. Experimental results show that compared with other mainstream methods, this paper still has good advantages in completing missing point clouds on the MVP dataset, and can obtain more complete point clouds.

#### 4.2.2. Mvp Dataset Robustness Test

In order to further verify the robustness of the method proposed in this paper, robustness experiments were also conducted on the MVP dataset. This article selected three categories—Airplane, Guitar, and Skateboard—from the MVP dataset and conducted incomplete point cloud prediction error testing. Table 7 shows the experimental results of the method proposed in this article.

The experimental results are shown in Table 7. When the Missing Ratio is 25%, the missing rates for the three are 0.813/0.573, 0.704/0.564, and 0.833/0.678, respectively. When the Missing Ratio is 50%, the missing rates for the three are 0.826/0.594, 0.682/0.559, and 0.875/0.664, respectively. When the Missing Ratio is 75%, the missing rates for the three are 0.833/0.612, 0.715/0.597, and 0.804/0.697, respectively. In the MVP dataset, the average input error for 25% is 0.783, the average input error for 50% is 0.794, and the average input error for 75% is 0.784. The input errors for all three parts are around 0.79. This also indicates that the network in this article has strong robustness in handling point clouds with different degrees of missing data, and can stably recover the missing point clouds.

#### 4.2.3. Mvp Comparison Experiment of Dataset Cd

**Figure 6:**
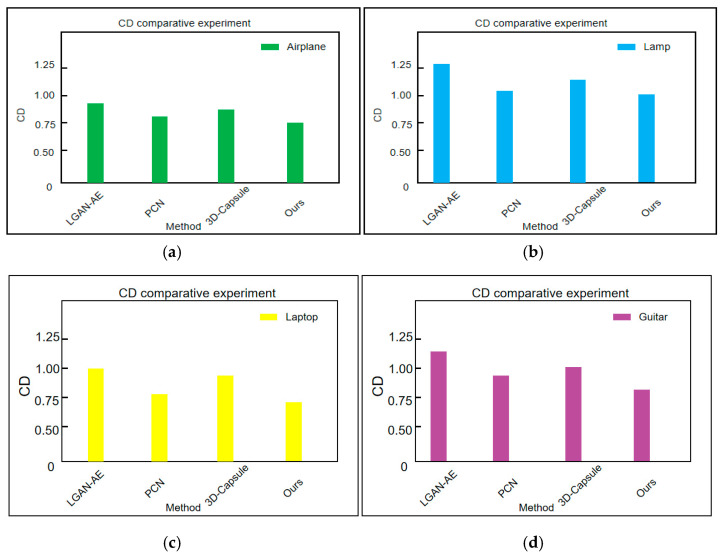
Mvp dataset CD comparative experiment. (**a**) Airplane CD comparative experiment. (**b**) Lamp CD comparative experiment. (**c**) Laptop CD comparative experiment. (**d**) Guitar CD comparative experiment.

This paper also conducted CD comparison experiments on the Airplane and Lamp categories in the MVP dataset. Figure 6 shows the experimental results. A larger CD indicates that the difference between the two sets of point clouds is greater. The smaller the distance, the better the reconstruction effect.

To further validate the effectiveness of the proposed method, this paper conducted CD comparison experiments on the Airplane, Lamp, Laptop, and Guitar categories of the Shapenet Part dataset. The experimental results are shown in Figure 6. Compared with LGAN-AE, the CD values of the proposed method in this paper decreased by 0.23, 0.14, 0.16, and 0.22 in the categories of Airplane, Lamp, Laptop, and Guitar, respectively; Compared to PCN, the CD values decreased by 0.06, 0.05, 0.05, and 0.08, respectively; Compared to 3D capsules, the CD values decreased by 0.22, 0.07, 0.18, and 0.12, respectively. These data show that the method proposed in this article significantly outperforms other mainstream methods in terms of CD values, with average reductions of 19%, 6%, 15%, and 15%, respectively.

#### 4.2.4. Mvp Dataset Ablation Experiment

To avoid the limitations of a single dataset, a series of ablation experiments were also conducted on the MVP dataset. These experiments systematically removed or replaced the SCMP and SEP-Net modules to observe and analyze changes in model performance, thereby accurately evaluating the contribution of each module. The ablation studies provide valuable guidance for further optimizing the model architecture, ensuring the model’s outstanding performance on the ShapeNet-Part dataset. The results are shown in Table 8.

As shown in Table 8, the proposed modules all contribute to a reduction in the Chamfer Distance (CD) value. When using only the SCMP module, the CD value decreases by 0.013; when using only the SEP-Net module, it decreases by 0.017. When both modules are used together, the CD value decreases by 0.028. Therefore, the SCMP and SEP-Net modules proposed in this paper have been experimentally verified to reduce the CD value by approximately 6%. The proposed method also effectively reduces geometric information loss, enhances feature representation capability, and generates more complete point clouds on the MVP dataset.

### 4.3. Prediction Error Training Experiment

**Figure 7:**
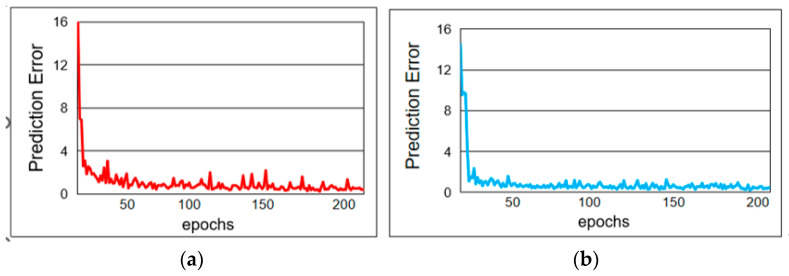
Training of dataset prediction error model. (**a**) Shapenet-Part training model. (**b**) MVP training model.

The point cloud completion prediction error experiment is a key step in evaluating the performance of point cloud completion algorithms. The error size is determined by quantifying the difference between the completed point cloud and the true complete point cloud. During the experiment, incomplete point cloud data must be prepared as input, and corresponding real and complete point cloud data should be obtained as a reference. Then, using the point cloud completion algorithm to be evaluated, the incomplete point clouds are completed to obtain the predicted point cloud data. Finally, by calculating the evaluation index values, the prediction error is quantified, and the algorithm’s performance is evaluated. The prediction error training in this article is shown in Figure 7.

Figure 7 shows the overall error value variation of the FFPF-Net method on the Shapenet-Part and MVP datasets after 200 rounds of training. From Figure 7a, it can be concluded that after 200 rounds of training, the training error value for Shapenet-Part is generally stable at around 0.2. From Figure 7b, it can be concluded that after 200 rounds of training, the MVP training error value is generally stable at around 0.25. Therefore, the method proposed in this article can stably perform tasks, and our method can converge quickly and stably in the early stage. During the process, the fluctuation amplitude is small, indicating strong stability. The results prove that the FFPF-Net method has both accuracy and stability.

### 4.4. Inference Time and Model Size

In the comprehensive evaluation system of artificial intelligence models, model performance is not solely defined by accuracy indicators. For point cloud processing, its data naturally has the characteristics of high dimensionality and uneven sampling density, and the core application scenarios generally have rigid constraints such as “limited hardware resources” and “zero tolerance for response delay”. This further amplifies the importance of inference time and model size. The following is a discussion on the model in this article.

As shown in Table 9, based on the mainstream experimental configuration in the field of point cloud processing (input point cloud size of 2048 sampling points, hardware platform unified as NVIDIA RTX 2080Ti GPU, Intel i7-10700K CPU), combined with the core structural characteristics of each model and experimental data in public literature, the inference time and model size (parameter quantity) are analyzed and compared. The FFPF Net model has a size of 2.8 M and an inference time of 6.5 ms. Compared to PCN, LGAN-AE, and 3D Capsule, the FFPF Net model has only slightly higher parameter count (2.8 M) and inference time (6.5 ms) than the minimalist PCN model, avoiding a significant decrease in efficiency caused by complex modules in LGAN-AE and 3D Capsule. The 2.8 M parameter count and 6.5ms inference time make it adaptable to scenarios with limited hardware resources, making it more practical for deployment compared to LGAN-AE (3.0 M/9.8 ms) and 3D Capsule (11.5 M/18.3 ms). The above comparison fully demonstrates that FFPF Net effectively controls the model size and inference time while ensuring completion accuracy, making it a reasonable design that balances performance and efficiency.

### 4.5. Point Cloud Visualization

**Figure 8:**
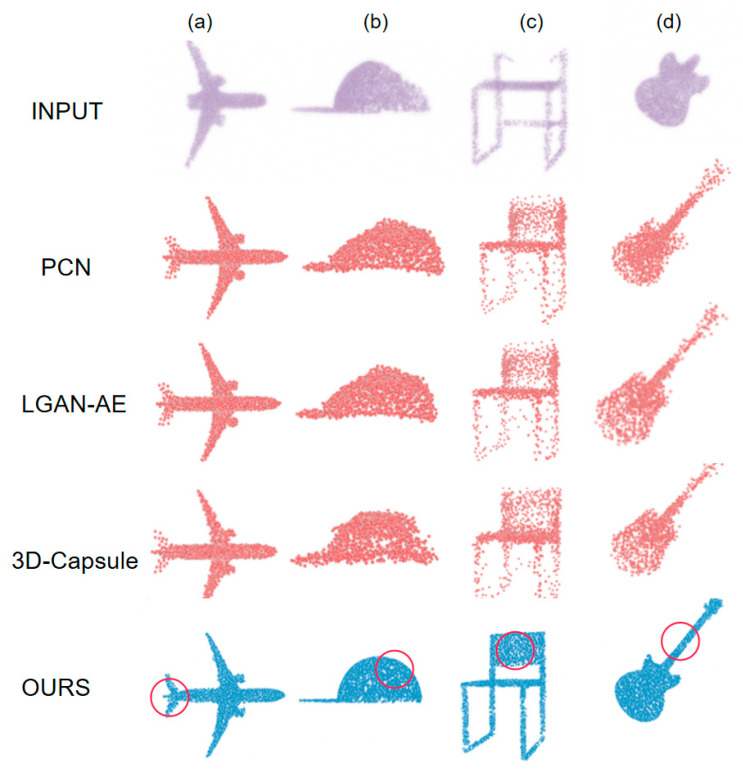
Point cloud visualization. (**a**) Airplane point cloud completion. (**b**) Cap point cloud completion. (**c**) Chair point cloud completion. (**d**) Guitar point cloud completion.

In order to gain a more intuitive understanding of the shape of point clouds and evaluate the completion effect, this paper conducted point cloud visualization experiments, presenting point cloud data in the form of three-dimensional graphics through visualization methods. The results are shown in Figure 8. Through visualization, we can clearly observe the shape, distribution, and surface features of point clouds, which is crucial for understanding the structure of point cloud data and evaluating the effectiveness of point cloud completion. By comparing the visualization results, we can intuitively evaluate the performance and effectiveness of completion algorithms, providing strong support for subsequent algorithm optimization and improvement.

As shown in Figure 8, compared with PCN, LGAN-AE, and 3D Capsule, the method proposed in this paper exhibits significant advantages in all categories. In the case of the aircraft shown in Figure 8a, the tail is completely missing from the input point cloud. PCN, LGAN-AE, and 3D Capsule cannot provide sufficiently complete reconstruction, while the proposed method more accurately restores the tail. For the hat depicted in Figure 8b, the upper right corner of the input point cloud is clearly missing. The completion of PCN, LGAN-AE, and 3D Capsule appears relatively rigid, while the proposed method better restores the original details of the cap. In Figure 8c, the backrest of the chair is clearly incomplete in the input point cloud. The results of PCN, LGAN-AE, and 3D Capsule are quite sparse, while the proposed method effectively restores the structural details of the backrest. For the guitar shown in Figure 8d, the upper part of the input point cloud is missing. The completion of PCN, LGAN-AE, and 3D Capsule lacks density, but the proposed method successfully restores the fine details of the guitar. In summary, the method proposed in this article has demonstrated excellent performance and broad application potential in point cloud completion. It better integrates object features, effectively restores missing details, and generates more complete, accurate, and realistic point clouds, providing a more solid data foundation for subsequent point cloud processing tasks.

## 5. Discussion

In response to the common limitations of insufficient capture of local structural features and weak control of fine-grained features in existing mainstream point cloud completion methods, this paper proposes two core modules (SCMP and SEP-Net) to address these deficiencies in a targeted manner. Among them, the SCMP module adopts a multi-level and multi-resolution farthest point sampling, local grouping, and Composite Multi-Layer Perceptron architecture. Firstly, key points are filtered through FPS; then K-nearest neighbors (KNN) are used to divide local regions; and finally multi-scale features are extracted through MLP and pooling, breaking through the limitations of traditional methods such as PCN and LGAN-AE that overly focus on global features. It can flexibly integrate local geometric details such as edges and textures of point clouds with global semantics, avoiding high-frequency geometric information omission. Finally, the dual-input encoding of “original point cloud + downsampling point cloud” is used to further preserve structural clues and provide high-quality feature input for subsequent completion. The SEP-Net module integrates “squeeze excitation” and pooling operations, encoding channel global information through global average pooling (Squeeze), learning channel weights through dual fully connected layers (Excitation), and adjusting feature responses through scale calibration. It can achieve adaptive recognition and enhancement of key channel features, significantly improving the network’s sensitivity to important features while suppressing redundant feature interference, with almost no increase in computational cost. Compared to traditional attention methods, it focuses more on “channel level feature calibration” and improves fine-grained structure reconstruction more directly.

This work focuses on the SCMP and SEP-Net modules, constructing an end-to-end architecture of “feature extraction–feature optimization–gradual generation,” which offers significant advantages in feature utilization and completion logic. However, the method proposed in this article heavily relies on predefined key point selection and other steps. Although it can effectively improve the processing performance of common structured data, in cases where the point cloud is severely missing or the shape is abnormally irregular, it may lead to the establishment of biased local features in subsequent parts, thereby limiting the overall performance of the model. Additionally, the improved performance achieved through multi-module collaboration inevitably increases model complexity. When training data is insufficient or lacks diversity, the network may overfit to specific datasets or object categories, thus compromising its completion performance in unseen scenarios. Furthermore, although our model excels in feature utilization and completion, its primary focus remains on accuracy rather than efficiency. No specific optimizations have been made for inference speed; as a result, its computational cost and inference time may still be higher than those of the lightest point cloud processing networks. This could limit its applicability in high-real-time scenarios such as high-speed autonomous driving or real-time robotic decision-making. Finally, the absence of a hardware platform for real-world data acquisition confined our experiments to benchmark datasets. Consequently, while the model achieves strong performance on these datasets, its robustness against real-world impairments such as noise, occlusion, and uneven point density remains unvalidated.

Based on the in-depth analysis of the limitations of this study, our future work will prioritize the following research directions:

(1) Regarding the dependencies of predefined sampling, future research will focus on learnable deformable attention modules, enabling the model to dynamically determine the importance of various regions in the point cloud. When faced with severe missing or abnormal shapes, the model can intelligently focus on the core structural regions, thereby improving its generalization ability to complex situations.

(2) In response to the issues of model complexity and computational efficiency, future research will focus on techniques such as channel pruning and dynamic inference paths, in order to construct a dual-mode system that can switch in real time according to actual needs. This will enable the full model to be activated when high precision is required and switch to lightweight mode when real-time performance is required, thereby enhancing its application capabilities in high-real-time scenarios.

(3) In response to interference issues in the real world, future research will actively seek cooperation and build a real-time data collection platform to validate model performance in real-world environments. On the other hand, research on anti-interference strategies such as training noise simulation and outlier removal at the algorithm level can improve the reliability of the model in complex physical environments.

## 6. Conclusions

Point cloud completion, as a core task in computer vision and 3D data processing, faces significant challenges due to the unordered and sparse nature of point cloud data, as well as inevitable missing data caused by external factors during acquisition. These issues contribute to the complexity of the completion task. Existing mainstream point cloud completion methods tend to focus on extracting global features of objects, while the extraction of local features is relatively insufficient. Moreover, in the process of generating complete point clouds, object details are often overlooked, resulting in a lack of fine-grained features in the completed point clouds. These shortcomings lead to suboptimal accuracy and completeness in the final results. To address the above issues, this paper first designs a multi-level, multi-resolution farthest point sampling strategy called SCMP. After sampling key points, this module performs local grouping and feature extraction operations to preserve structural details and construct point cloud representations rich in local semantics, thus providing high-quality input for the subsequent completion process. In addition, this paper introduces SEP-Net, a strategy that combines channel attention mechanisms with pooling operations. By suppressing redundant features and enhancing key responses, SEP-Net refines feature representation and significantly improves the network’s ability to reconstruct fine-grained structures, thereby enhancing the overall completion performance. Based on improvements in local feature extraction and detailed structure modeling, this paper proposes a point cloud completion network called FFPF-Net, which integrates the SCMP module and SEP-Net architecture. Extensive experiments were conducted on the ShapeNet-Part and MVP datasets. Results show that the proposed method improves the average prediction accuracy by 1.3 and 1.4 compared to L-GAN and PCN, respectively. In robustness testing, the average Chamfer Distance was 0.783 on the ShapeNet-Part dataset and 0.824 on the MVP dataset, indicating that the proposed method significantly enhances the level of detail in point cloud completion. These improvements demonstrate the potential of the method for broader application in various 3D scenarios.

## Figures and Tables

**Table 1:** Shapenet-Part dataset the overall point cloud completion result.

Category Method	LGAN-AE (Pred→ GT/GT→ Pred)	PCN (Pred→ GT/GT→ Pred)	3DCapsule (Pred→ GT/GT→ Pred)	CGCN (Pred→ GT/GT→ Pred)	DAF-SAC (Pred→ GT/GT→ Pred)	Ours (Pred→ GT/GT→ Pred)
Airplane	0.856/0.722	0.800/0.800	0.826/0.881	0.432/0.427	0.325/0.316	**0.273/0.248**
Bag	3.102/2.994	2.954/3.063	3.228/2.722	1.785/1.674	1.532/1.314	**0.946/0.792**
Cap	3.530/2.823	3.466/2.674	3.439/2.844	2.784/2.476	2.351/2.224	**1.266/1.189**
Car	2.232/1.687	2.324/1.738	2.503/1.913	2.013/1.476	1.521/1.487	**0.601/0.435**
Chair	1.541/1.473	1.592/1.538	1.678/1.563	1.465/1.437	1.078/1.024	**0.490/0.457**
Guitar	0.394/0.354	0.367/0.406	0.298/0.461	0.321/0.306	**0.241**/**0.214**	0.401/0.396
Lamp	3.181/1.918	2.757/2.003	3.271/1.912	1.887/1.804	1.563/1.464	**1.123/0.664**
Laptop	1.206/1.030	1.191/1.155	1.276/1.254	0.846/0.766	0.423/0.456	**0.323/0.256**
Motorbike	1.828/1.455	1.699/1.459	1.591/1.664	0.784/0.702	0.654/0.621	**0.543/0.398**
Mug	2.732/2.946	2.893/2.821	3.086/2.961	1.054/1.104	0.984/1.354	**0.762/0.749**
Pistol	1.113/0.967	**0.968**/**0.958**	1.089/1.086	1.232/1.122	1.023/1.054	1.114/1.342
Skateboard	0.887/1.023	0.816/1.206	0.897/1.262	0.423/0.412	0.365/0.302	**0.268/0.202**
Table	1.694/1.601	1.604/1.790	1.870/1.749	1.452/1.322	0.941/0.842	**0.553/0.442**
Mean	1.869/1.615	1.802/1.662	1.927/1.713	1.268/1.156	1.001/0.975	**0.579/0.475**

**Table 2:** Shapenet-Part dataset missing point cloud completion result.

Category Method	LGAN-AE (Pred→ GT/GT→ Pred)	PCN (Pred→ GT/GT→ Pred)	3DCapsule (Pred→ GT/GT→ Pred)	CGCN (Pred→ GT/GT→ Pred)	DAF-SAC (Pred→ GT/GT→ Pred)	Ours (Pred→ GT/GT→ Pred)
Airplane	3.357/1.130	5.060/1.243	2.676/1.401	1.457/1.321	1.315/1.046	**1.123/1.101**
Bag	5.707/5.303	3.251/4.314	5.228/4.202	3.675/3.644	**3.231**/**3.047**	3.949/3.823
Cap	8.968/4.608	7.015/**4.240**	11.04/4.739	6.413/6.326	**4.551**/4.264	5.304/4.875
Car	4.531/2.518	2.741/2.123	5.944/3.508	2.623/2.456	2.537/2.087	**2.522/1.873**
Chair	7.359/2.339	3.952/2.301	3.049/2.207	2.665/2.637	2.278/2.114	**2.113/1.843**
Guitar	0.838/0.536	1.419/0.689	0.625/0.662	0.523/0.446	**0.441**/0.454	0.465/**0.431**
Lamp	8.464/3.627	11.61/7.139	9.912/5.847	6.877/6.524	5.533/5.024	**5.132/3.471**
Laptop	7.649/1.413	3.070/1.422	2.129/1.733	2.046/2.016	1.433/1.241	**1.273/1.011**
Motorbike	4.914/2.036	4.962/1.922	8.617/2.708	3.185/2.812	2.254/2.121	**2.232/1.782**
Mug	6.139/4.735	3.590/3.591	5.155/5.168	4.210/4.074	3.954/3.374	**3.143/3.243**
Pistol	3.944/1.424	4.484/1.414	5.980/1.782	1.473/1.323	1.213/1.104	**1.122/1.055**
Skateboard	5.613/1.683	3.025/1.740	11.49/2.044	2.015/1.751	1.457/**1.302**	**1.143**/1.358
Table	2.658/2.484	2.503/2.452	3.929/3.098	2.874/2.432	**2.041/1.932**	2.754/1.956
Mean	5.395/2.603	4.360/2.661	5.829/3.008	3.079/2.905	**2.480**/2.239	2.482/**2.140**

**Table 3:** Incomplete point cloud prediction error.

Category Missing Ratio	25% (Pred→ GT/GT→ Pred)	50% (Pred→ GT/GT→ Pred)	75% (Pred→ GT/GT→ Pred)
Airplane	**0.784**/0.520	**0.763**/0.531	**0.792**/0.594
Guitar	**0.683**/0.493	**0.668**/0.547	**0.694**/0.552
Skateboard	**0.803**/0.658	**0.824**/0.603	**0.795**/0.656

**Table 4:** Shapenet-Part dataset ablation experiment.

SCMP	SEP-Net	CD
×	×	0.300
√	×	0.289
×	√	0.263
√	√	**0.245**

**Table 5:** MVP dataset the overall point cloud completion result.

Category Method	LGAN-AE (Pred→ GT/GT→ Pred)	PCN (Pred→ GT/GT→ Pred)	3DCapsule (Pred→ GT/GT→ Pred)	CGCN (Pred→ GT/GT→ Pred)	DAF-SAC (Pred→ GT/GT→ Pred)	Ours (Pred→ GT/GT→ Pred)
Airplane	1.754/1.956	1.653/1.745	1.934/1.776	0.874/0.727	0.651/0.606	**0.542/0.764**
Bed	4.397/5.023	4.654/4.956	5.203/4.971	2.478/2.421	2.351/2.343	**2.321/2.341**
Watercraft	3.486/3.234	3.789/3.643	3.764/3.547	1.851/1.846	1.325/**1.354**	**1.231**/1.452
Car	4.653/4.983	4.134/4.857	4.784/5.021	3.541/3.426	2.911/**2.487**	**2.584**/3.471
Chair	2.354/2.763	2.764/2.657	2.546/2.835	2.315/2.437	2.078/2.034	**1.851/1.942**
Guitar	0.767/0.754	0.781/0.895	0.754/0.902	0.323/0.306	0.241/0.224	**0.234/0.214**
Lamp	4.385/2.964	4.746/5.312	4.432/4.674	1.658/1.634	1.543/1.574	**1.421/1.512**
Laptop	1.206/**1.030**	**1.093**/1.342	1.283/1.443	1.081/1.266	1.223/1.256	1.324/1.241
Motorbike	2.354/2.221	2.365/2.431	2.112/2.435	1.875/1.712	1.553/1.532	**1.413/1.531**
Sofa	3.423/3.134	3.541/3.462	3.123/3.431	2.784/2.604	2.584/2.346	**2.512/2.131**
Pistol	1.453/**1.342**	**1.433**/1.578	1.453/1.586	1.334/1.526	1.523/1.563	1.471/1.571
Skateboard	1.293/1.245	1.283/1.256	1.238/1.245	1.201/1.184	1.085/0.862	**0.841/0.681**
Table	3.845/2.927	3.875/3.901	3.523/3.741	2.684/2.322	2.651/2.642	**2.314/2.615**
Mean	2.721/2.583	2.778/2.926	2.781/2.893	1.846/1.801	1.671/**1.601**	**1.543**/1.651

**Table 6:** MVP dataset missing point cloud completion result.

Category Method	LGAN-AE (Pred→ GT/GT→ Pred)	PCN (Pred→ GT/GT→ Pred)	3DCapsule (Pred→ GT/GT→ Pred)	CGCN (Pred→ GT/GT→ Pred)	DAF-SAC (Pred→ GT/GT→ Pred)	Ours (Pred→ GT/GT→ Pred)
Airplane	4.331/1.453	5.132/1.674	3.613/2.371	1.952/1.874	1.652/1.646	**1.562/1.423**
Bed	9.714/10.065	8.314/8.921	10.512/9.524	6.871/6.634	5.762/5.036	**5.326/4.952**
Watercraft	7.892/7.471	8.424/8.214	7.934/7.621	4.414/4.024	4.021/3.781	**3.624/3.596**
Car	5.213/3.971	3.413/2.967	6.313/5.023	3.658/3.416	**2.631**/2.477	3.532/**2.461**
Chair	8.962/4.281	4.721/3.651	4.301/3.134	4.535/4.137	3.045/3.124	**2.781/2.073**
Guitar	1.213/1.112	1.984/1.545	1.034/1.157	1.033/1.016	**0.784/0.854**	1.114/1.201
Lamp	9.345/5.821	12.941/8.412	10.462/6.984	7.213/7.024	6.543/6.014	**6.041/4.786**
Laptop	8.515/5.023	4.142/2.768	3.614/3.081	2.135/2.025	**1.435/1.311**	1.981/1.861
Motorbike	6.051/3.571	5.812/2.614	9.531/4.671	4.652/4.522	4.157/4.101	**3.795/2.945**
Sofa	7.324/6.724	7.624/6.962	6.731/6.782	5.820/5.774	5.652/5.378	**5.532/5.342**
Pistol	5.158/2.076	5.714/**2.061**	7.084/3.515	2.653/2.603	2.492/2.434	**2.412**/2.301
Skateboard	7.795/2.963	4.658/**2.856**	13.724/4.071	3.457/3.321	3.057/3.009	**2.614**/3.014
Table	4.625/4.672	4.681/4.423	4.784/4.424	4.672/3.933	**4.031**/3.751	4.831/**3.615**
Mean	6.626/4.554	5.966/4.390	6.895/4.811	4.080/3.869	3.481/3.301	**3.473/3.040**

**Table 7:** MVP dataset incomplete point cloud prediction error.

Category Missing Ratio	25% (Pred→ GT/GT→ Pred)	50% (Pred→ GT/GT→ Pred)	75% (Pred→ GT/GT→ Pred)
Airplane	**0.813**/0.573	**0.826**/0.594	**0.833**/0.612
Guitar	**0.704**/0.564	**0.682**/0.559	**0.715**/0.597
Skateboard	**0.833**/0.678	**0.875**/0.664	**0.804**/0.697

**Table 8:** MVP dataset ablation experiment.

SCMP	SEP-Net	CD
×	×	0.372
√	×	0.359
×	√	0.342
√	√	**0.314**

**Table 9:** Inference time and model size.

Method	Param (M)	Time (ms)
PCN	1.2	4.2
FFPF-Net	2.8	6.5
LGAN-AE	3.0	9.8
3D-Capsule	11.5	18.3

## Data Availability

The data presented in this study are available on request from the corresponding author.
